# Calcium electroporation and electrochemotherapy for cancer treatment: Importance of cell membrane composition investigated by lipidomics, calorimetry and *in vitro* efficacy

**DOI:** 10.1038/s41598-019-41188-z

**Published:** 2019-03-18

**Authors:** K. L. Hoejholt, T. Mužić, S. D. Jensen, L. T. Dalgaard, M. Bilgin, J. Nylandsted, T. Heimburg, S. K. Frandsen, J. Gehl

**Affiliations:** 1 Center for Experimental Drug and Gene Electrotransfer, Department of Oncology, Copenhagen University Hospital Herlev, Herlev, Denmark; 2 Niels Bohr Institute, University of Copenhagen, Copenhagen, Denmark; 3 Department of Science and Environment, Roskilde University, Roskilde, Denmark; 4 Danish Cancer Society Research Center (DCRC), Copenhagen, Denmark; 5 Center for Experimental Drug and Gene Electrotransfer, Department of Clinical Oncology and Palliative Care, Zealand University Hospital, Roskilde, Denmark; 6 Department of Clinical Medicine, Faculty of Health and Medical Sciences, University of Copenhagen, Copenhagen, Denmark

## Abstract

Calcium electroporation is a novel anti-cancer treatment investigated in clinical trials. We explored cell sensitivity to calcium electroporation and electroporation with bleomycin, using viability assays at different time and temperature points, as well as heat calorimetry, lipidomics, and flow cytometry. Three cell lines: HT29 (colon cancer), MDA-MB231 (breast cancer), and HDF-n (normal fibroblasts) were investigated for; (a) cell survival dependent on time of addition of drug relative to electroporation (1.2 kV/cm, 8 pulses, 99 µs, 1 Hz), at different temperatures (37 °C, 27 °C, 17 °C); (b) heat capacity profiles obtained by differential scanning calorimetry without added calcium; (c) lipid composition by mass spectrometry; (d) phosphatidylserine in the plasma membrane outer leaflet using flow cytometry. Temperature as well as time of drug administration affected treatment efficacy in HT29 and HDF-n cells, but not MDA-MB231 cells. Interestingly the HT29 cell line displayed a higher phase transition temperature (approximately 20 °C) versus 14 °C (HDF-n) and 15 °C (MDA-MB231). Furthermore the HT29 cell membranes had a higher ratio of ethers to esters, and a higher expression of phosphatidylserine in the outer leaflet. In conclusion, lipid composition and heat capacity of the membrane might influence permeabilisation of cells and thereby the effect of calcium electroporation and electrochemotherapy.

## Introduction

Electroporation describes the use of brief electric pulses to transiently permeabilise cell membranes allowing uptake of otherwise impermeant molecules^1^. The common feature of electroporation-based therapies is permeabilisation of the cell membrane by application of electrical pulses thereby inducing an electric field that exceed the transmembrane potential of the plasma membrane.

Electroporation based therapies, utilized as anticancer treatments, include gene therapy^2,3^, irreversible electroporation^4^, and electrochemotherapy^5,6^.

In electrochemotherapy tumor cells are permeabilised by electroporation thereby enhancing their uptake of chemotherapeutic drugs (primarily bleomycin and cisplatin are used)^1^. Electrochemotherapy is currently in use in many cancer centers as a safe and efficient treatment of cutaneous and subcutaneous metastases^7–10^. The use of electrochemotherapy for treatment of internal tumors is currently being investigated in clinical trials^11–16^.

Calcium electroporation is a novel anti-cancer treatment where supraphysiological doses of calcium are internalized by electroporation causing cell death^17^. The usage of calcium instead of chemotherapeutic drugs presents several advantages: it is non-mutagenic, has a long durability, medical professionals other than oncologists can administer it, and setting aside the cost of electrodes and electroporator it is inexpensive^17–20^. The low cost of treatment, provided affordable electrodes and electroporator are available, is specifically advantageous considering that up to 80% of cancers occur in low-income and middle-income countries^21^. Preclinical investigations of calcium electroporation suggest that calcium electroporation causes cell death associated with acute ATP-depletion^17,22^ and that the treatment can be performed using the same electroporation parameters as applied in electrochemotherapy^23^ and other electroporation parameters^24^. Importantly calcium electroporation, like electrochemotherapy, shows a difference in sensitivity between normal and malignant cells *in vitro*^25–27^ and *in vivo*^28^, which might be explained in part by differences in membrane repair capacities^29^. The first clinical trial on calcium electroporation was recently published^20^ with studies on head and neck cancer and keloids now completed.

The time of pore closure after electroporation has been investigated *in vitro*^30–32^ and *in vivo*^33^ and a window of uptake both pre- and post-pulse is described for several molecules including CrEDTA^34^ and bleomycin^5^ but not for calcium. Permeability of a membrane describes the membrane’s ability to allow passage of molecules from the exterior to the interior of the membrane and vice versa. The plasma membrane is a dynamic structure and its permeability is affected by membrane composition, temperature, and the concentrations of among others anesthetics and calcium^35,36^. Calcium is known to also affect resealing of a damaged membrane^37,38^ and to influence the melting transition of cell membranes^35^. As calcium electroporation may find importance in the clinic, we decided to investigate optimal temperature and time of administration of calcium in relation to electroporation, comparing with bleomycin electroporation as control.

We therefore designed a study to investigate (1) the optimal time of calcium addition relative to electroporation in three different cell lines, (2) a possible temperature dependence, and (3) characteristics of these cell lines using lipid composition analysis by mass spectrometry (lipidomics), differential scanning calorimetry, and flow cytometric analysis.

As described, we compared calcium electroporation to bleomycin electroporation to investigate possible differences in the dependency of drug administration before and after electroporation as well as temperature. Moreover, the combination of calcium and bleomycin was investigated to examine if addition of calcium would change the effect of bleomycin, as evidenced by alteration of the cytotoxic effect of bleomycin.

## Results

### Influence of temperature and time of drug administration on calcium electroporation, bleomycin electroporation and calcium-bleomycin electroporation

**Figure 1:**
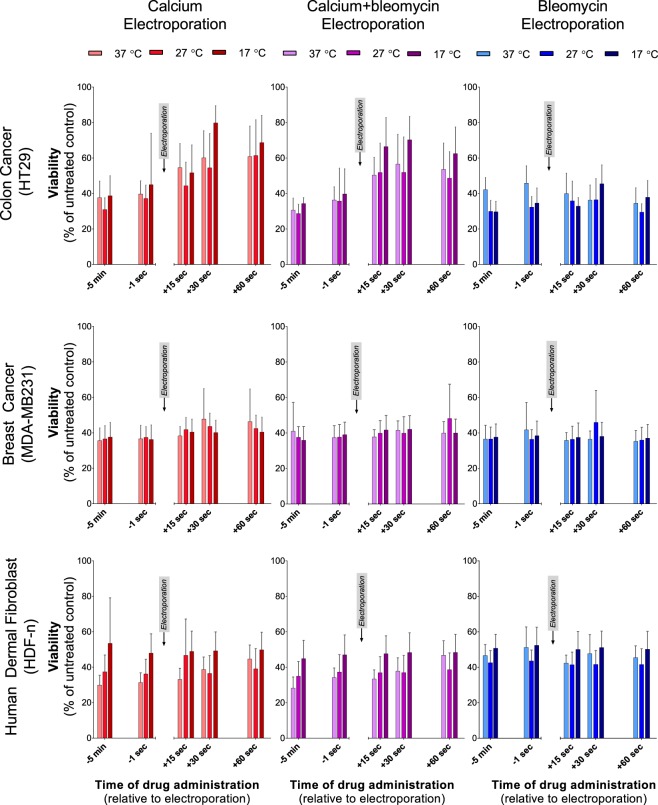
Electroporation treatment of three cell lines. HT29 (human colon cancer; top panel), MDA-MB231 (human breast cancer; middle panel) and HDF-n (human dermal fibroblast; bottom panel) treated with calcium electroporation (left), calcium-bleomycin electroporation (middle), and bleomycin electroporation (right). Drug was added at different time points relative to electroporation and experiments were performed at different temperatures (37 °C, 27 °C, and 17 °C). On the HT29 cell line treatment effect of calcium electroporation or calcium-bleomycin electroporation was dependent on temperature and time of drug administration whereas treatment effect of bleomycin electroporation was independent on time of addition but showed dependency on temperature when bleomycin was added before electroporation. Treatment at 27 °C resulted in highest treatment effect for this cell line. Treatment of MDA-MB231 showed neither temperature nor time of addition dependency for any of the treatments investigated. On the HDF-n cell line treatment effect of calcium electroporation or calcium-bleomycin electroporation was dependent on time of drug administration and on temperature whereas treatment effect when treating with bleomycin electroporation was independent on temperature and time of drug administration. Treatment at 37 °C resulted in lowest survival for this cell line. Results from control samples are shown in Supplementary Fig. S1. Mean + SD, n = 5–8, each performed as individual experiments. Obvious outliers have been removed from the data set. Statistical comparisons (two-way ANOVA) are described in the results section.

Each cell line was treated with calcium electroporation and bleomycin electroporation. As an effect of calcium on the permeability of the membrane, this could potentially alter bleomycin toxicity, why the combination of both drugs with electroporation (calcium-bleomycin electroporation) was also investigated (Fig. 1).

Drugs were added at five different time points relative to electroporation, which took place at 37 °C, 27 °C and 17 °C. Because the cells were only exposed to experimental temperatures for 5 minutes prior to treatment, the effect of temperature on the lipid metabolism of the cells is disregarded. Calcium was added to a final concentration of 5 mM and bleomycin to a concentration of 10 µM, which are doses used in the literature for *in vitro* studies^17,23^. The treatment effect was assessed by measuring cell viability and a low viability equaled a high treatment effect. Results from four different negative control conditions are shown in Supplementary Fig. S1. Limited effects were observed after treatment with drug alone or electroporation alone compared with untreated controls in all cell lines at all tested temperatures. Treatment with calcium alone resulted in 88–119% viability, treatment with calcium and bleomycin resulted in 86–113% viability, treatment with bleomycin alone resulted in 80–104% viability, and electroporation alone resulted in 70–88% viability, which correlate with previous studies on these cell lines^39^.

#### The colon cancer cell line (HT29)

Figure 1 (top, left graph) shows results from treatment of the HT29 cell line with calcium electroporation demonstrating that treatment efficacy was influenced by temperature and time of calcium addition relative to electroporation.

A dependency on time of calcium addition was observed for calcium electroporation at all three temperatures. When adding calcium 5 minutes before electroporation treatment effect was significantly greater than when adding calcium 30 or 60 seconds after electroporation regardless of treatment temperature being 37 °C (p < 0.05), 27 °C (p < 0.05), or 17 °C (p < 0.0001).

A statistically significant difference in treatment effect between the 3 temperatures was only found at one of the investigated time points; addition of calcium at 30 seconds after electroporation resulted in treatment effect that was significantly lower at 17 °C than at 27 °C (p < 0.05). Importantly, there was no difference in treatment effect between the 3 temperatures, when calcium was added before electroporation (Fig. 1, top, left graph).

The effect of bleomycin electroporation (Fig. 1, top, right graph) was only significantly influenced by time of drug administration, when bleomycin was added 30 seconds after electroporation instead of 5 minutes before at 17 °C (p < 0.05). Surprisingly, when adding bleomycin 5 minutes before electroporation, treatment effect was significantly lower at 37 °C than at both 27 °C and 17 °C (p < 0.005).

Treatment of the HT29 cell line with calcium-bleomycin electroporation (Fig. 1, top, middle graph) generated results similar to those from treatment with calcium electroporation showing that time of drug administration and temperature influenced treatment efficacy. Addition of calcium and bleomycin 5 minutes before electroporation resulted in significantly higher treatment effect than addition of calcium and bleomycin at 15, 30, or 60 seconds after electroporation regardless of treatment temperature being 37 °C (p < 0.005), 27 °C (p < 0.05), or 17 °C (p < 0.0001).

#### The breast cancer cell line (MDA-MB231)

The treatment effect on the MDA-MB231 cell line was independent of both temperature and time of drug administration for calcium electroporation, bleomycin electroporation, and calcium-bleomycin electroporation (Fig. 1, middle panel).

#### The normal dermal fibroblast cell line (HDF-n)

Results from treatment of the HDF-n cell line with calcium electroporation (Fig. 1, bottom, left graph) showed that treatment effect was dependent on temperature and, at 37 °C also on time of calcium administration. At 37 °C, the treatment effect decreased significantly when calcium was added 60 seconds after electroporation compared to addition of calcium 5 minutes before electroporation (p < 0.05). When calcium was added before electroporation (5 minutes before or 1 second before) the treatment effect was significantly higher at 37 °C than at 17 °C (p < 0.05).

Treatment of the HDF-n cell line with bleomycin electroporation showed no dependency on temperature or time of drug administration (Fig. 1, bottom, right graph).

When the normal cell line was treated with calcium-bleomycin electroporation (Fig. 1, bottom, middle graph) a dependency on temperature and, at 37 °C also on time of drug administration was observed similar to those seen with calcium electroporation. At 37 °C, treatment effect again decreased significantly when calcium and bleomycin was added 60 seconds after electroporation compared to addition of calcium and bleomycin 5 minutes before electroporation (p < 0.0001). A significant difference in treatment efficacy between 37 °C and 17 °C, with 37 °C resulting in the highest cell kill, was observed when adding drugs 5 minutes before (p < 0.01), or 15 seconds after (p < 0.05) electroporation.

### Investigations of cell membrane heat capacity and lipid composition

As the cell lines reacted differently to changes in temperature and time of calcium addition and as we hypothesized this difference to be caused by differences in cell membrane characteristics, we wanted to further investigate the cell membranes of the three cell lines. Thus, the following experiments were performed on the plasma membranes of the three cell lines: a) differential scanning calorimetry (DSC) to investigate the change in heat capacity of the cell membranes in response to changes in temperature, b) lipidomics performed with mass spectrometry to investigate differences in the lipid composition of the cell membranes, c) flow cytometry to investigate the exposure of phosphatidylserine in the outer leaflet of the cell membranes.

#### Differential scanning calorimetry (DSC)

The assumption is that a maximum in the heat capacity of the cell membrane is correlated with a high lateral compressibility^36,40^, which may facilitate electropore formation in the cells. If a pore is created, its environment is compressed and work is performed. In the absence of an electrical field, the free energy ∆F (work) of pore formation is given by

$${\rm{\Delta }}F=\frac{1}{2}\cdot \frac{1}{{\kappa }_{T}^{A}A}{\rm{\Delta }}{A}^{2}$$

where ${\kappa }_{T}^{A}$ is the area compressibility, A is the area of the membrane, and ∆A is the area of the pore. This implies that the free energy of the pore is lower when the area compressibility is high and it is easier to create a pore^41,42^. A cell membrane can be described as having two temperature dependent phases, (1) a gel-like phase (low permeability) and (2) a fluid phase (permeability higher than in the gel-phase). The two phases are separated by a phase transition^42^ in which the plasma membrane reaches its maximum permeability^42–44^. The transition is an order-disorder transition within the lipid membranes and not a transition of the cells as a whole^40,42^.

**Figure 2:**
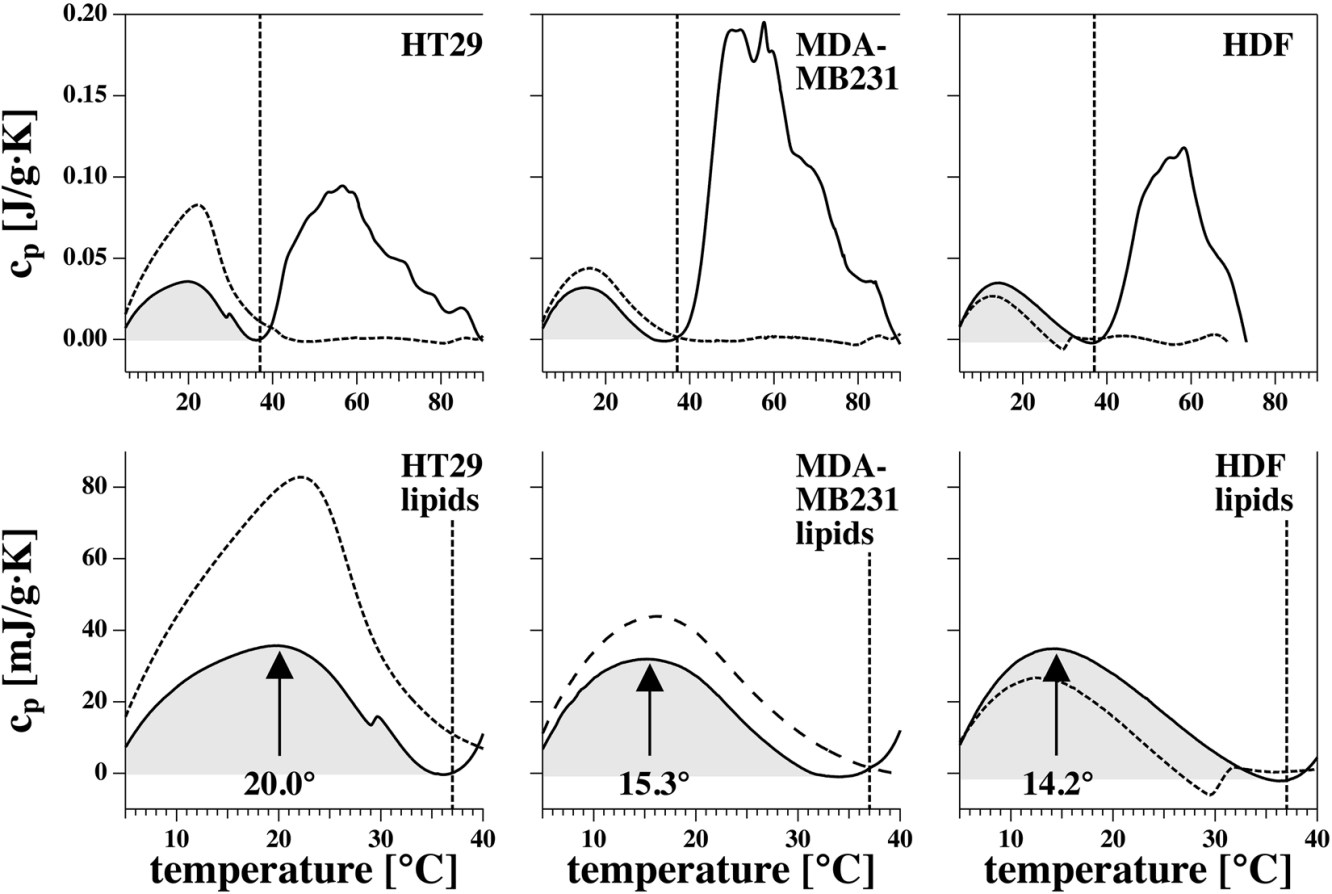
Heat capacity profiles of the three investigated cell lines. Heat capacity profiles of HT29 (colon cancer cell line; left), MDA-MB231 (breast cancer cell line; middle), and HDF-n (normal dermal fibroblast cell line; right). Solid lines represent the first scan over the full temperature range (0–90 °C). Dashed lines represent the second calorimetric scan over the full temperature range. The protein peaks have disappeared (because the proteins have denaturated in the first scan over the full range of temperatures) while the lipid peaks remain. The amplitude of the lipid peaks can change between scans due to subtraction of base line but the position of the peak (temperature) is more constant between scans. The vertical line represents the physiological temperature. Bottom panel: enlargement of the top panel from 0–40 °C, where approximate transition temperatures are indicated.

All three cell lines displayed these phases (Fig. 2), but interestingly the HT29 cell line displayed a higher phase transition temperature (approximately 20 °C) versus MDA-MB231 (approximately 15 °C) and HDF-n (approximately 14 °C). This means that the phase transition of the colon cancer cell line occurred within the temperature range investigated (17–37 °C) meaning that between the temperatures investigated this cell line underwent changes in plasma membrane permeability.

The difference between the heat capacity profiles indicated that the three cell lines have cell membranes of different composition, which would affect the cell membranes’ reactions to changes in temperatures and to electroporation. This may be part of the explanation for the differential effects of calcium electroporation observed between the three cell lines in electroporation experiments. Though the phase transition temperature of the HDF-n and MDA-MB231 cell line were similar, only the MDA-MB231 cell line was independent of both temperature and time of drug administration. This indicates that numerous factors influence permeability of cell membranes and further studies are warranted to elucidate possible explanations.

The effect of calcium electroporation around the transitioning temperature on the colon cancer cell line was investigated, too. (Further details in Supplementary Data 2 and Supplementary Fig. S2).

#### Lipidomics: Membrane composition

**Figure 3:**
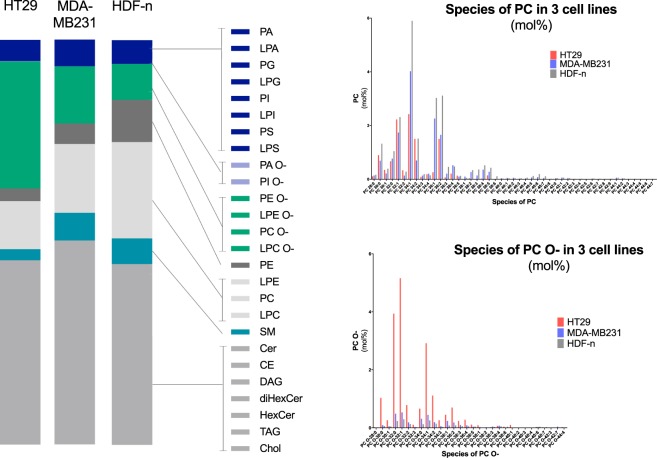
Lipidomics. The left panel displays the distribution of lipid classes in the membranes of three cell lines: HT29 (colon cancer), MDA-MB231 (breast cancer), and HDF-n (normal fibroblast cell line). Amounts are given in mol%. No difference in the content of negatively charged lipids (dark blue and light blue) was found between the cell membranes of the three cell lines. Neither did the membranes of the cell lines differ with regards to their content of phosphatidylserine (PS). HT29 showed much higher levels of the lipids in ether form, e.g. phosphatidylcholine (PC) and phosphatidylethanolamine (PE) in their ether form (PC O- and PE O-) than MDA-MB231 and HDF-n. The right panel displays the content of species of PC in the plasma membranes of the three cell lines in the form of esters (PC; top panel) and in the form of ethers (PC O-; bottom panel). The experiment was independently repeated, confirming the initial result.

Mass spectrometry based lipid analysis was performed on preparations of plasma membranes (Fig. 3). The lipidomics data represented both the inner and outer membrane leaflet, thus we did not have information of the distribution of the lipids between the two leaflets.

The cellular lipodome of the three cell lines was compared focusing on differences with regards to levels of phosphatidylserine (PS) and other negatively charged lipids, since calcium can bind to these lipids^45,46^, but no differences were found between the three cell lines (Fig. 3, left panel – light and dark blue).

The only remarkable difference between the investigated normal cell line HDF-n and the two investigated malignant cell lines HT29 and MDA-MB231 were the content of phosphatidylethanolamine (PE) where the normal cell line HDF-n contained more PE than the two malignant cell lines HT29 and MDA-MB231 (Fig. 3, left panel – brown), however the significance of this is to our knowledge not previously investigated and further studies are warranted.

Interestingly, one factor separated the colon cancer cell line from the breast cancer cell line and the normal cell line. The colon cancer cell line showed much higher levels of the lipids in ether form, e.g. phosphatidylcholine (PC) and phosphatidylethanolamine (PE) in their ether form (PC O- and PE O-) than the two other cell lines (Fig. 3, left panel – green). As ethers are synthesized from esters the cell line consequently has lower levels of these lipids in ester form (Fig. 3, left panel – light grey). PC O- and PE O- are not negatively charged, but their structure is believed to contribute to more membrane stiffness^34^ and a higher threshold of electropermeabilization^35^ than their counterparts containing ester-links^47,48^. This may contribute to the dependency of temperature and time of drug administration observed in treatment of the colon cancer cell line with calcium electroporation.

#### Flow cytometric analysis of expression of phosphatidylserine in the outer leaflet of the cell membrane

Flow cytometric analysis was performed to investigate if any differences existed between the three cell lines in their exposure of PS in the outer leaflet of their plasma membranes. For each cell line untreated cells were stained with Annexin V-FITC and PI. The Annexin V stain is much used to detect apoptosis; however here we investigated untreated cells, under the assumption that the PS observed in the outer membrane leaflet was not a result of apoptosis but related to the composition of the cell membrane. Concomitant PI staining was used to indicate cell death.

**Figure 4:**
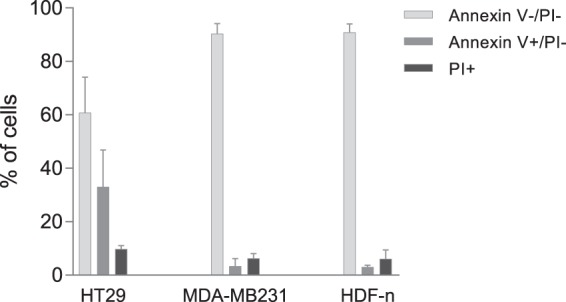
Flow cytometric analysis of phosphatidylserine (PS) exposure. Percentage of PS-exposing cells in the outer leaflet of the plasma membrane in each cell line. Untreated cells were stained with propidium iodide (PI) and Annexin V before flow cytometric analysis. The Annexin V stain is much used to detect apoptosis; however here we investigated untreated cells under the assumption that the PS observed in the outer leaflet was not a result of apoptosis but related to the composition of the cell membrane. Thus, Annexin V+/PI− show the percentage of cells exposing PS in the outer membrane. PI+ show the percentage of dead cells. Mean + SD, n = 3–4, each performed as individual experiments.

Figure 4 presents the distribution between Annexin V−/PI− cells, PI+ cells, and Annexin V+/PI− cells in all three cell lines and representative dot-plots are displayed in Supplementary Fig. S3. PI-positive cells were considered damaged cells with compromised membrane integrity (no statistical significant difference between cell lines). Subsequently only Annexin V+/PI− cells were considered positive for exposure of PS in the outer leaflet of their cell membranes. Thus, the number of PS exposing cells may actually be higher than what we report as the damaged cells that were excluded from the count of PS exposing cells, may have exposed PS in the outer leaflet of their membrane. Staining of the HT29 cell line resulted in a significantly higher percentage of Annexin V+/PI− cells compared to MDA-MB231 and HDF-n cells (p < 0.05). Calcium has previously been shown to interact with PS in a cell membrane^46^ thereby stabilizing the membrane^45^. It is therefore interesting that a high amount of HT29 cells expose PS in its outer membrane leaflet and that this cell line was sensitive to changes in time of calcium addition during the calcium electroporation process. For the HT29 cell line 33.1% of cells were Annexin V+/PI− showing that one third of the HT29 cells exposed PS in the outer leaflet of their cell membranes. This indicates a level of heterogeneity in composition of cell membranes in the HT29 cell line.

Untreated samples of the MDA-MB231 cell line and the HDF-n cell line had a percentage of PS-positive cells of approximately 3% (MDA-MB231 3.35% and HDF-n 3.07%; no significant difference). Thus, the amount of MDA-MB231 and HDF-n cells exposing PS in the outer leaflet of the cell membranes was significantly lower than the HT29 cells (p < 0.05). Only 6–10% of cells were PI positive (dead cells) and no significant difference were observed between cell lines.

## Discussion

In this study, we have investigated permeabilization efficacy (determined by cell death due to calcium or bleomycin internalization) and dependence on temperature and time of drug administration (pre or post pulse). These results have been supplemented with data from lipidomics analyses, differential scanning calorimetry, and analysis of the outer membrane leaflet by flow cytometry, and a hitherto unveiled heterogenity between membranes of cell lines was observed, which might affect the sensitivity to electroporation-based therapies.

The results evidence that temperature has a significant influence on the effect of calcium electroporation and for the HT29 cell line also on the effect of bleomycin electroporation. The effect of temperature on electropermeabilization has previously been described by Kanduser *et al*.^49^ and an increase in temperature has recently been shown to increase gene expression following gene electrotransfer in addition to permit a decrease in the electrical field applied in gene electrotransfer *in vivo*^50^. The observed differences in effect of electroporation-based treatments might be more pronounced with higher temperature differences between samples. For the HT29 and the HDF-n cell line treatment effect of calcium-bleomycin electroporation was similar to that of calcium electroporation. This similarity in results between the two treatments could indicate that calcium, when combined with bleomycin and electroporation, may alter the degree or duration of the permeabilized state, and may in this way alter intracellular uptake of bleomycin.

The heat capacity profile of the HT29 cell line might contribute to the explanation for the temperature and time of addition dependent results of calcium electroporation and calcium-bleomycin electroporation. Permeability of a lipid membrane is highest around the transition phase of that membrane, and the temperature at which this transition occurs, is influenced by, among others, the concentration of calcium and anesthetics. Anesthetics lower the transition temperature whereas calcium elevates it^35,36,45,51^. The effect of calcium on the heat capacity profile and hence on the permeability of a cell membrane may in part explain why treatment with calcium electroporation and calcium-bleomycin electroporation was not conclusively optimal at 21 °C; calcium results in a shift of the heat capacity curve towards higher temperatures meaning that the temperature resulting in the phase transition of the HT29 cell membrane was elevated and approaching 27 °C and that the permeability of the cell membrane at 17 °C was lowered. This was corroborated by the data from treatment of the HT29 cell line at 21 °C. This temperature seemed to be optimal for bleomycin electroporation but not for calcium electroporation and calcium-bleomycin electroporation (Supplementary Data 2, Supplementary Fig. S2).

As for the two other cell lines, the heat capacity profiles did not exhibit transition within the temperature range investigated. However, the effect of calcium on the heat capacity profiles is not investigated in this study and further studies to evaluate the effect of calcium on the heat capacity profiles of the cell lines are warranted. In the presence of calcium, any phase transition will occur at a higher temperature and this may explain why the effect of calcium electroporation and calcium–bleomycin electroporation on the HDF-n cell line was highest at 37 °C. The heat capacity of the MDA-MB231 cell line also displayed distinct phases, and the fact that treatment of this cell line was entirely independent of changes in temperature therefore cannot be explained by its heat capacity profile.

As the composition of a membrane highly determines its permeability, we also investigated the plasma membranes of the three cell lines using lipidomics. A remarkable difference in the content of lipids in ether form was found between the three cell lines with the HT29 cell line containing a much higher level of the lipids PE and PC in the form of ethers than the other two cell lines. The structure of ethers favors more dense packing than that of esters and studies on vesicles have shown that the threshold for electropermeabilization increases when the ratio of ethers to esters increases^47,48^. Under conditions used in the clinic, i.e. adding calcium prior to electroporation, the toxicity of calcium electroporation and calcium-bleomycin electroporation was similar for all three cell lines. However, when changing the temperature or adding calcium after electroporation the viability of the HT29 cell line was particularly affected. The sensitivity of this cell line to a degradation of treatment conditions may be due to an underlying lower membrane permeability of this cell line, and further studies may elucidate other influencing elements.

No difference in the total content of PS was found between the three cell lines in lipidomics, which however only provided information on the *total* distribution of lipids in the plasma membrane and did not distinguish between the inner and outer membrane leaflet.

One factor that is known to generally differ between normal and malignant cells is the fraction and localization of the negatively charged phospholipid PS^52^. Plasma membrane asymmetry is an important feature of normal cells, in which PS is almost exclusively localized in the inner leaflet of the cell membrane, unless the cells are undergoing apoptosis, where PS is relocated to the outer leaflet^53^. In many malignant cells this plasma membrane asymmetry is lost resulting in the localization of PS in the outer membrane leaflet, also when the cells are not undergoing apoptosis^54–57^. Calcium is known to interact with PS thereby stabilizing the cell membrane^45,46^ and exposure of PS in the outer cell membrane leaflet may therefore influence the effect of calcium electroporation. A very small amount of the normal dermal fibroblasts was found to expose PS in its outer cell membrane leaflet. This was also the case for the breast cancer cells, however 33.1% of the colon cancer cells exposed PS in the outer leaflet of their cell membranes. Thus, on the cell line with the highest amount of cells exposing PS in the outer leaflet, there was a difference in treatment effect of calcium electroporation and calcium-bleomycin electroporation between administration of calcium pre- or post-pulses regardless of temperature. A similar time of addition dependency was not observed on the other two cell lines or on the HT29 cell line with bleomycin electroporation. It could be speculated 1) that calcium when added before electroporation alters the degree or duration of the permabilized state on the HT29 cell line and 2) that the electrical pulses ameliorate this proposed stabilizing effect of calcium on the HT29 cell membrane, due to the higher PS-exposure, but further studies are warranted.

In the investigated normal cell line only a few cells were positive for PS-exposure; however treatment of this normal cell line with calcium electroporation and calcium-bleomycin electroporation at 37 °C resulted in dependence of time of drug addition. As this particular normal cell line is known to have a high membrane repair capacity^29^, the dependence on time of calcium addition in calcium electroporation and calcium-bleomycin electroporation at 37 °C may be related to efficient membrane repair in this cell line.

The influence of temperature and time of calcium administration on the effect of calcium electroporation has not previously been investigated and its determination is important in the clinic as it might lead to optimization of the treatment. Though an exact temperature control is difficult to obtain in the clinic, the cooling effect of cold anesthetics and cold calcium can be circumvented by letting the drugs normalize to room temperature prior to injection. Calcium and anesthesia have contrasting effects on the melting transition of cell membranes and the combined effects of these drugs on the permeability of cell membranes would be very interesting to investigate.

In conclusion, we have shown that temperature and time of calcium administration influence the effect of calcium electroporation; and importantly that this effect is cell line dependent. We found marked differences in the composition of membranes in these cell lines using lipidomics analyses, in particular a high concentration of ether lipids in the cell line showing the most dynamic response with regards to temperature dependency. Furthermore, difference in lipids in the outer leaflet of the cell membrane may influence the level of permeabilization. As calcium is a cation that may interact with lipids in the outer membrane leaflet, the influence of outer leaflet lipid composition on the level of permeabilization may in particular pertain to calcium electroporation. This study indicates that membrane composition might influence the effect of electroporation based therapies. Other factors of cell membrane composition than those investigated in this study may also affect sensitivity of treatment and further studies are warranted.

## Materials and Methods

### Cell culture

Two human cancer cell lines and a normal human cell line were used: HT29 (ATCC #HTB-38), a human colorectal adenocarcinoma, MDA-MB231 (ATCC #HTB-26), a human breast adenocarcinoma, and HDF-n, a normal neonatal primary dermal fibroblast (kindly provided by Dr. Marie-Pierre Rols, Institute of Pharmacology and Structural Biology, IPBS, Toulouse, France) for which passages were limited to 10. HT29 was grown in RPMI-1640 culture medium (Gibco, Invitrogen) and MDA-MB231 and HDF-n were grown in DMEM culture medium (Gibco, Invitrogen). The culture medium for all cell lines was added 10% fetal calf serum (Gibco, Invitrogen), penicillin (100 U/ml) and streptomycin (100 µg/ml). The cells were grown in an incubator at 37 °C with 5% CO_2_. All cell lines tested negative for mycoplasma.

Authentication: The cancer cell lines were authenticated by short tandem repeat profiling (LGC Standards) which showed perfect match for the HT29 cell line and a match in 7 out of 9 profile loci (loci D7 and VWA had lost a peak) for the MDA-MB231 cell line and these two profile changes have been assessed not to influence the results of these experiments.

### Electroporation experiments

After trypsination and wash, cells were suspended in HEPES buffer (10 mM HEPES (Lonza), 250 mM sucrose, and 1 mM MgCl_2_ in sterile water).

A volume of 270 µl of cell suspension (6.1 × 10^6^ cells/ml) was added to a cuvette containing either 30 µl of HEPES or 30 µl of drug dissolved in HEPES (final concentration of cells 5.5 × 10^6^ cells/ml) and incubated for 5 min in a heating block (Grant, QBD2) at either 17 °C, 21 °C, 27 °C, or 37 °C before electroporation. Temperature of the samples was measured and was within +/−5 °C of the temperature of the heating block with 17 °C and 37 °C having the largest variations. Electroporation took place in 4 mm cuvettes with aluminium electrodes (Molecular BioProducts, Inc.), and pulses were delivered by a square wave electroporator (BTX T820) with the following electroporation parameters: 8 pulses of 99 µs, 1 Hz, and 1.2 kV/cm (applied voltage to electrode distance ratio). Drugs were dissolved in HEPES buffer and added to a final concentration of: CaCl_2_ 5 mM, and bleomycin (Bleomycin Baxter) 10 µM, before or after electroporation. Both doses were chosen as it was found in initial studies^17,23,26^ that they result in more than 80% cell kill when combined with electroporation. For samples where drug was added after electroporation, cells were electroporated with HEPES buffer followed by addition of 50 µl drug dissolved in HEPES buffer at the designated time. No mixing of drug and sample was done before or after electroporation, and drugs were added by placing the pipette at the bottom of the cuvette. The added volume before electroporation constituted 10% of the total volume of the sample. Thus, 30 µl drug or HEPES buffer and 270 µl cells were added to the cuvette followed by electroporation. If drug was added after electroporation this was done in a volume of 50 µl at the designated time. In samples where drug was added before electroporation, 50 µl HEPES buffer was added before transferring to medium to secure the same cell concentration in all samples. All samples were incubated at the designated temperature for 5 min before electroporation, independent on time of drug addition. Prior to addition to cell suspension drugs were kept at room temperature.

Samples were divided into 5 groups according to the time point at which drug was added relative to electroporation: 5 min before electroporation, 1 s before electroporation, 15 s after electroporation, 30 s after electroporation, or 60 s after electroporation. Each group contained 3 samples: one treated with calcium electroporation, one treated with bleomycin electroporation, and one treated with calcium and bleomycin combined with electroporation (calcium-bleomycin electroporation). An additional group consisted of 5 control samples: calcium, bleomycin, calcium and bleomycin (all three without electroporation), electroporation without drug, and an untreated control.

After electroporation or no electroporation, samples were incubated for 15 min at 37 °C and 5% CO_2_ in the cuvettes before transfer to 5 ml culture medium. Then, 100 µl were seeded in a 96 well plate (3.1 × 10^4^ cells per 100 µl). MTS assay was performed 21–25 hours after treatment using a microplate reader (Synergy HT, BioTek) and Gen5 microplate reader software (BioTek).

### Heat capacity of cell membranes

Differential scanning calorimetry (DSC) was performed from membrane preparations of the three cell lines. Cells were continually harvested and frozen at −80 °C until a sufficient amount for DSC was obtained.

Membranes were prepared from thawed samples. Lysis buffer (Natriumchlorid 137 mM, Tris 20 mM, Nonidet P40 1%, Glycerol 10%, PMSF Sigma P7626 1 mM, AProtinin Sigma A6103 10 µg/ml, Natrium metavanadat Sigma 59008 0.5 mM, Leupeptin Sigma L2882 1 µg/ml) was added to each cell pellet. Cells were transferred to 2 ml Eppendorf tubes containing a steel ball and mechanically homogenized for 2 min at 20 Hz using a Tissue Lyser (QIAGEN). Cells were then centrifuged (VWR Microstar 17R) at 300 g for 10 min causing precipitation of the nuclei. The supernatant was then centrifuged (VWR Microstar 17R) at 3000 g for 10 min causing precipitation of mitochondria. The supernatant was lastly centrifuged (Sigma 3K20) at 40,000 g for 1.5 hours causing formation of plasma membrane pellets. All centrifugations took place at 4 °C. Membrane pellets were resuspended in PBS (0.7 ml). Membrane preparations were kept at 4 °C for 1–3 days until analysis by heat calorimetry.

Differential scanning calorimetry: Samples were degassed under high vacuum. Calorimetric scans were performed on a Malvern MicroCal VP-DSC (Northampton, MA) differential scanning calorimeter. Heating rates were kept at 20 °C/hour, filtering period was 5 seconds and feedback was set to none. PBS buffer was used as a reference. Each scan was run on 0.5152 ml of sample (which is the size of the calorimeter cell) containing between 12 and 32 mg of dry matter. The weight of substance was measured by drying the sample under air stream and high vacuum. First, samples were equilibrated in one or two scans from 0–38 °C, to avoid any differences due to the time samples were kept at 4 °C. These scans were discarded. Then, the full range scans were performed. No signal averaging or other forms of data smoothing were applied to the thermograms. The data was processed using IgorPro. Between scans of different samples, calorimetric cells were thoroughly cleaned with 37% HCl.

### FACS

Expression of phosphatidylserine (PS) in the outer leaflet of the cell membrane was investigated by flow cytometry using Mebcyto Apoptosis kit (MBL, #4700).

Untreated cells in HEPEs buffer (500,000 cells in 90 μl) were centrifuged at 1000 rpm for 3 min, the supernatant was removed and cells were resuspended in 85 µl binding buffer (part of the kit).

Annexin V-FITC (10 µl) and propidium iodide (PI, 5 µl, 100 µg/ml) were added and samples were incubated in the dark at room temperature for minimum 15 min. Then, 400 µl binding buffer was added to all samples and transferred to FACS tube before flow cytometric analysis at room temperature using FACS Calibur (Becton Dickinson) counting 10,000 events for each sample.

Unstained, only Annexin V stained and only PI stained samples of all three cell lines were used to adjust the settings of the instrument for each cell line. Each sample was run in duplicates or triplicates and the assay was performed three-four times for each cell line, thus n = 3–4. For the data analyses, the gating was set so all events were selected except the debris. The events were divided in 4 quadrants with respectively PI-positive vs PI-negative and Annexin V-positive vs Annexin V-negative based on the control samples (unstained, only Annexin V stained and only PI stained). The percentages of gated events were used for the statistical analyses and graphs. PI staining indicate cell death. Representative dot-plots are displayed in Supplementary Fig. S3.

### Lipidomics

Lipidomics was performed on membrane preparations. Cell cultures were harvested after growth at 37 °C. Membranes were prepared as described in the above section of heat calorimetric experiments to collect plasma membranes. After preparation, the membrane pellets were frozen in liquid nitrogen and stored at −80 °C.

#### Two-step Lipid Extraction

75ul of cell membrane lysates were subjected to lipid extraction by a modified two-step Bligh and Dyer protocol executed on ice^58,59^. Briefly, the sample aliquots were spiked with 10 µl internal lipid standard mix containing 30 pmol CE 15:0-D7, 20 pmol Cer 18:1;2/12:0;0, 200 pmol Chol-D4, 10 pmol DAG 12:0/12:0, 20 pmol diHexCer 18:1;2/12:0;0, 25 pmol HexCer 18:1;2/12:0;0, 25 pmol LPA 17:0, 20 pmol LPC 10:0, 25 pmol LPE 13:0, 15 pmol LPG 17:1, 20 pmol LPI 13:0, 20 pmol LPS 17:1, 25 pmol PA 12:0/12:0, 20 pmol PC-OO 18:1/18:1, 25 pmol PE 12:0/12:0, 15 pmol PG 12:0/12:0, 15 pmol PI 8:0/8:0, 20 pmol PS 12:0/12:0, 20 pmol SHexCer 18:1;2/12:0;0, 20 pmol SM 18:1;2/12:0;0, 10 pmol TAG 17:0/17:0/17:0, and 20 pmol triHexCer 18:1;2/17:0;0. Samples were extracted with 1000 µl chloroform/methanol (10:1, v/v) and mixed at 2000 rpm for 15 min at 4 °C. The samples were centrifuged for 2 min at 2000 g to facilitate phase separation. The lower organic phase 10:1 was subsequently transferred to a new tube for 30 min vacuum evaporated. The remaining aqueous upper phase were reextracted with 1000 µl chloroform/methanol (2:1, v/v) and mixed at 2000 rpm for 15 min at 4 °C. The lower organic phase 2:1 was transferred to the evaporated 10:1 extract tube and vacuum evaporated for 60 min. The combined lipid extracts were dissolved in 100 µl chloroform/methanol (1:2, V/V).

#### Mass Spectrometry

Lipid extracts were subjected to mass spectrometric analysis using a Q Exactive mass spectrometer (Thermo Fisher Scientific, Waltham, MA, USA) equipped with a TriVersa NanoMate (Advion Biosciences, Ithaca, NY, USA). 10 µl aliquots of lipid extracts were loaded in 96-well plates, mixed with 12.9 µl of 13.3 mM in 2-propanol for positive or 10 µl of 0.2% methyl amine chloroform/methanol (1:5, V/V) for negative ion mode analysis, respectively. Samples were infused using a back pressure of 1.25 psi and ionization voltage of 0.95 kV for positive mode. For negative mode samples were infused using a back pressure of 0.7 psi and ionization voltage of −1.06 kV. The acquisition cycle consisted of FT MS and FT MS/MS scans in positive and negative ion mode.

FT MS spectra in positive mode were acquired in low *m/z* range of 400 to 730 and high *m/z* range of 575 to 1201 at the mass resolution of R_*m/z* 200_ = 140,000; automated gain control (AGC) value of 1 × 10^6^; three micro-scans; maximum injection time of 150 ms.

FT MS spectra in negative mode were acquired in low *m/z* range of 400 to 675 and high *m/z* range of 500 to 1202 at the mass resolution of R_*m/z* 200_ = 140,000; automated gain control (AGC) value of 1 × 10^6^; three micro-scans; maximum injection time of 150 ms.

Sequential FT MS/MS data were acquired using automated gain control (AGC) value of 1 × 10^5^; maximum injection time of 150 ms, one microscan, and R_*m/z* 200_ = 35,000.

FT MS/MS analysis performed in 1.0008 u steps across the m/z range 400.3–1000.8 using a quadrupole ion isolation width of 1.2 u and stepped nCE at 18, 28 and 38% for negative mode and for positive mode it was 15, 20 and 25%.

FT MS – selective ion monitoring (SIM) spectra in positive mode were acquired in *m/z* range of 396 to 416 at the mass resolution of R_*m/z* 200_ = 140,000; automated gain control (AGC) value of 1 × 10^6^; maximum injection time of 500 ms.

FT MS/MS – targeted parallel reaction monitoring (tPRM) spectra in positive ion mode were acquired for 2 scans, *m/z* 404.3887 and 408.4138, at the mass resolution of R_*m/z* 200_ = 35,000; automated gain control (AGC) value of 1 × 10^5^; maximum injection time of 500 ms, using a quadrupole ion isolation width of 1.2 u and stepped nCE at 13, 18 and 23%.

#### Annotation of Lipid Species

The annotation of lipid species was as previously described^59–61^. The glycerophospholipid and glycerolipid species annotated according to their sum composition: <lipid class> <total number of C in fatty acid moieties>:<total number of double bonds in fatty acid moieties> (e.g., PC 34:1). The sphingolipid species annotated according to their sum composition: <lipid class> <total number of C in the long-chain base and fatty acid moiety>:<total number of double bonds in the long-chain base and fatty acid moiety>;<total number of OH groups in the long-chain base and fatty acid moiety> (e.g., SM 34:1;2)^58,59^.

#### Data Processing, Lipid Identification and quantification

Lipid species detected by high resolution FT MS and FT MS/MS analysis were identified using LipidXplorer^62,63^, and quantified using Excel based calculation. In short, lipid species detected by FT MS and annotated by sum composition were quantified by normalizing their intensity to the intensity of an internal lipid standard of identical lipid class and multiplying with the spike amount of the internal lipid standard^59,64,65^.

### Statistics

In electroporation experiments, differences in viability between the investigated temperatures and time points for drug administration were assessed by two-way analysis of variance (ANOVA) using SPSS (version 19). The assumption of normally distributed data were tested using a combination of P-P plots, kurtosis and skewness, K-S test, and S-W test. In the event of a violation of this assumption, data were log transformed. For the assumption of homogeneity of variance, Levene’s test was used. In the event this assumption was violated, an adjustment was made using Welch’s F method. Post hoc analyses were conducted using a Bonferroni correction for multiple comparisons when assumptions for parametric testing were met. Games-Howell was used for post hoc analyses, where homogeneity of variance was violated. Differences in Annexin V and PI levels were assessed by one-way ANOVA using SAS (version 9.4). The assumption of normally distributed data were tested using P-P plots and for the assumption of homogeneity of variance, Levene’s test was used. No violation of the assumptions were seen. The alpha criteria for significance were set at 0.05 for all tests. Results are displayed in graphs as mean + 1 SD.

## Supplementary information

Dataset 1

## Data Availability

All data are available via contact to corresponding author.
