# Supplementary material for: Calcium electroporation and electrochemotherapy for cancer treatment: Importance of cell membrane composition investigated by lipidomics, calorimetry and in vitro efficacy
Source: Sci Rep. 2019 Mar 18;9:4758. doi: 10.1038/s41598-019-41188-z (PMC6427041; doi:10.1038/s41598-019-41188-z)
Supplement: Supplementary file 1 — Dataset 1 [file 41598_2019_41188_MOESM1_ESM.docx]

**Supplementary information**

**Calcium electroporation and electrochemotherapy for cancer treatment: Importance of cell membrane composition investigated by lipidomics, calorimetry and in vitro efficacy**

Hoejholt, K.L.^1^, Mužić, T.^2^, Jensen, S.D.^1^, Dalgaard, L.T.^3^, Bilgin, M.^4^, Nylandsted, J.^4^, Heimburg, T.^2^, Frandsen, S.K.^1,5*^, Gehl, J.^1,5,6*^

^1^Center for Experimental Drug and Gene Electrotransfer, Department of Oncology, Copenhagen University Hospital Herlev, Denmark
^2^Niels Bohr Institute, University of Copenhagen, Denmark

^3^Department of Science and Environment , Roskilde University , Roskilde , Denmark
^4^Danish Cancer Society Research Center (DCRC), Denmark 
^5^Center for Experimental Drug and Gene Electrotransfer, Department of Clinical Oncology and Palliative Care, Zealand University Hospital, Denmark
^6^Department of Clinical Medicine, Faculty of Health and Medical Sciences, University of Copenhagen, Denmark

^*^Corresponding Authors: Julie Gehl, Center for Experimental Drug and Gene Electrotransfer, Department of Clinical Oncology and Palliative Care, Zealand University Hospital, Denmark
Email: [kgeh@regionsjaelland.dk](mailto:kgeh@regionsjaelland.dk)

Stine Krog Frandsen, Center for Experimental Drug and Gene Electrotransfer, Department of Clinical Oncology and Palliative Care, Zealand University Hospital, Denmark

Email: stfra@regionsjaelland.dk

## Supplementary 1

(Supplementary to Figure 1)**
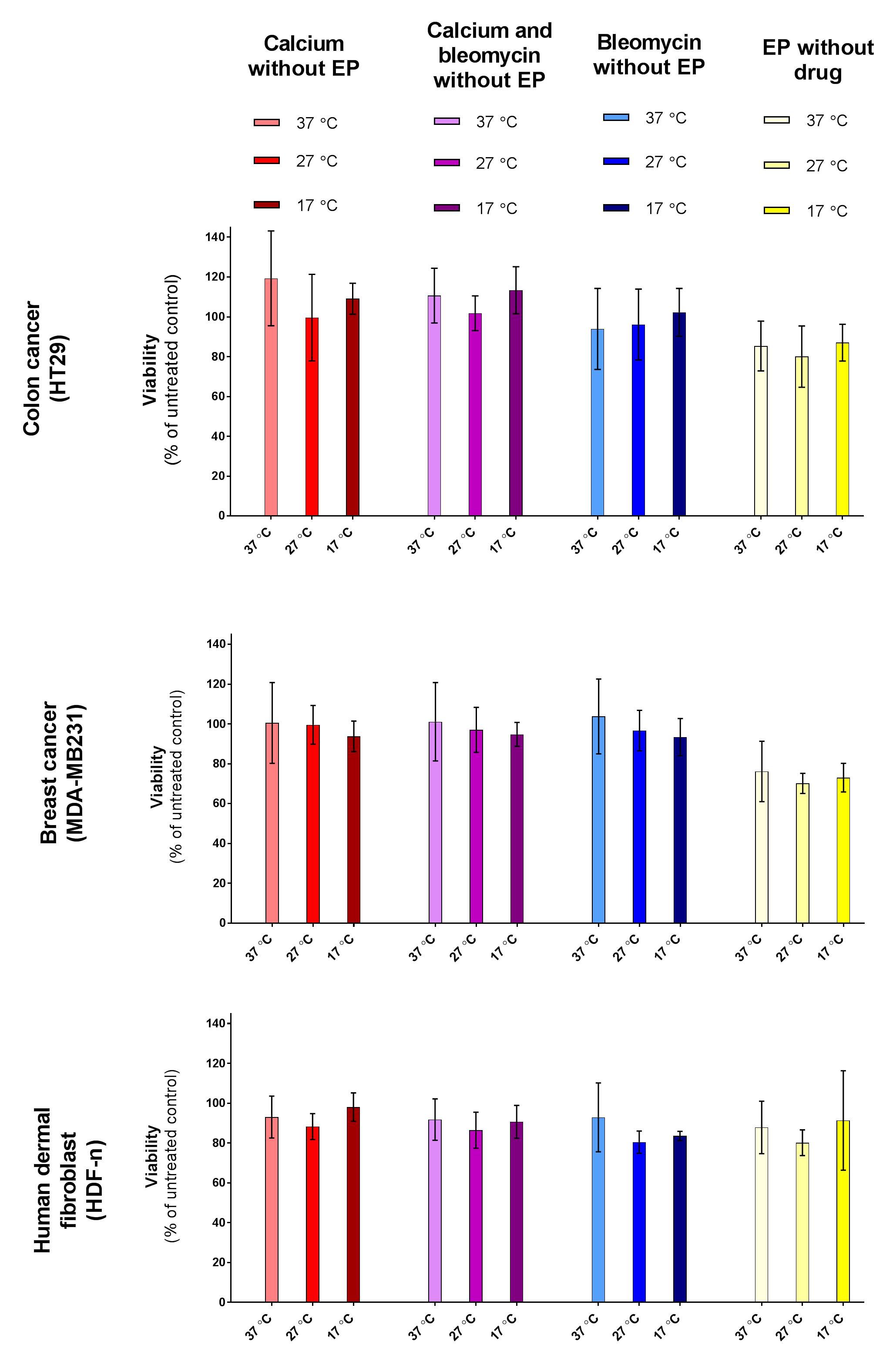
**

**Supplementary Figure S1: Viability of control samples treated without electroporation or without drugs**

*HT29 (human colon cancer; top panel), MDA-MB231 (human breast cancer; middle panel) and HDF-n (human dermal fibroblast; bottom panel) treated with calcium without electroporation, calcium and bleomycin without electroporation, bleomycin without electroporation and electroporation in buffer without added drug. Experiments were performed at different temperatures (37 °C, 27 °C, and 17 °C) and viability was measured one day after treatment using MTS assay. Viability is shown as percentage of untreated control. Mean+SD, n=7-8 performed as individual experiments.*

**Supplementary 2**

(Supplementary to Figure 2)

## *Supplementary Data 2: Electroporation experiments at 21 °C*

Since the HT29 cell line showed dependency of temperature and time of drug administration, this cell line was further investigated in electroporation experiments at the peak temperature determined from the DSC without calcium. The peak temperature is the temperature at which the permeability of the cell membrane reaches its maximum. In the absence of calcium, the temperature resulting in the highest permeability (the peak temperature) was defined from the heat calorimetric experiment to be 20 °C. This value is estimated to have an accuracy of +/-1 °C and further electroporation experiments were performed at 21 °C.

**
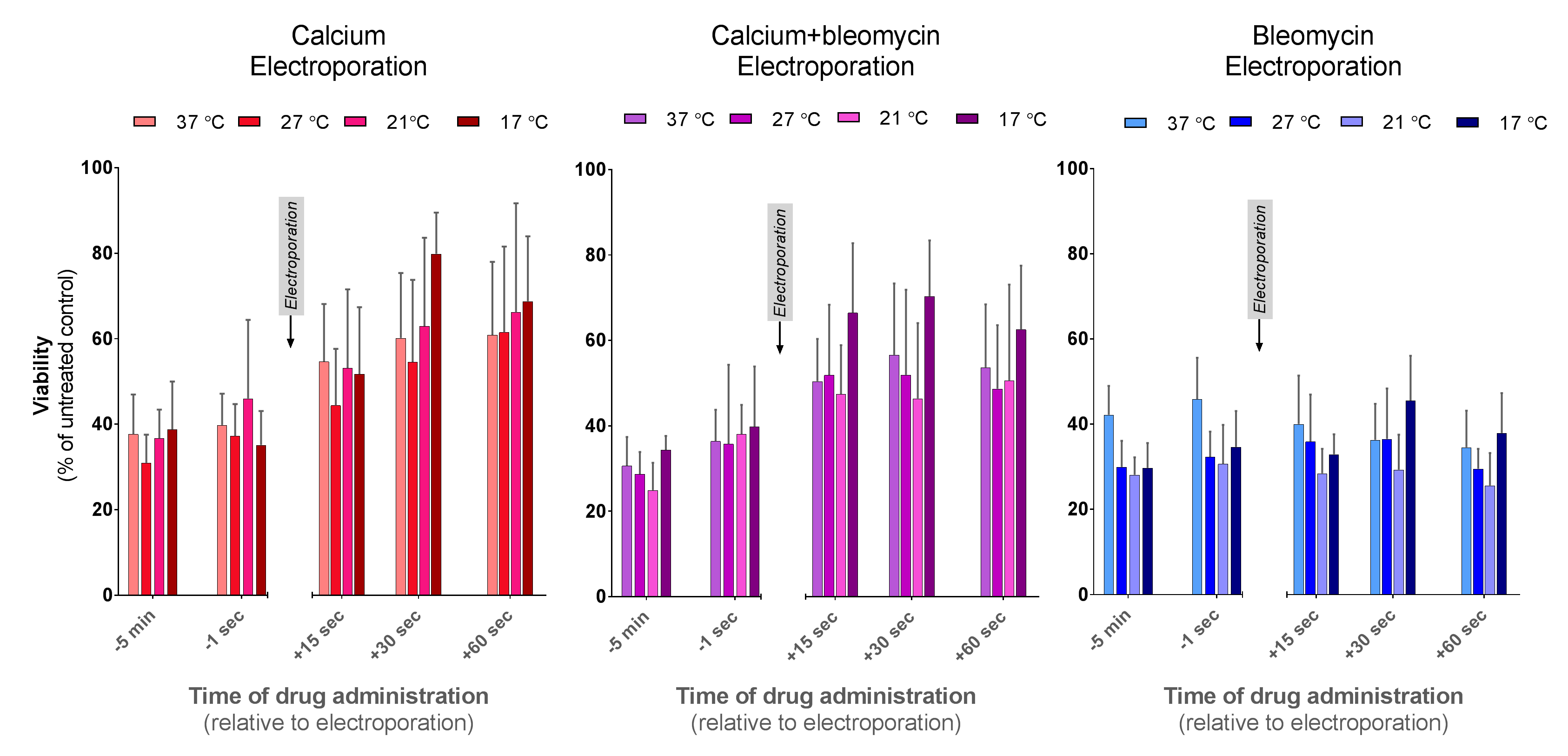
**

**Supplementary Figure S2: Electroporation at four temperatures, including peak temperature 21°C***HT29 (colon cancer cell line) treated with calcium electroporation (left), calcium-bleomycin electroporation (middle), and bleomycin electroporation (right) at four temperatures 37 °C, 27 °C, 21 °C, and 17 °C. Drugs were added at different time points relative to electroporation. Viability is shown as percentage of untreated control.
mean+SD. n=5-7 performed as individual experiments. Statistical comparisons (two-way ANOVA) are described in the section below.*

It would be predicted from previous literature (ref. 36, 42, 51) that the peak in heat capacity would represent the melting transition of the membrane and thus be the point where electroporation is putatively most efficient. Indeed we did see that the effect of bleomycin electroporation was optimal at 21 °C, and that when adding bleomycin 5 minutes before electroporation, treatment effect at 21 °C was significantly larger than at 37 °C (p<0.001). The effect of bleomycin electroporation when bleomycin was added 30 or 60 seconds after electroporation, was also significantly greater at 21 °C than at 17 °C (p<0.05).

The time of addition dependency observed when treating with calcium electroporation and calcium-bleomycin electroporation at 37 °C, 27 °C, and 17 °C was not statistically significant for calcium electroporation at 21 °C (p=0.058 and p=0.062 for addition of calcium respectively 30 seconds and 60 seconds after electroporation compared to addition 5 minutes before electroporation). In calcium-bleomycin electroporation at 21 °C adding drugs 5 minutes before electroporation resulted in statistically significant higher treatment effect than addition of drugs after electroporation (p<0.05)

Surprisingly, the effect of calcium-bleomycin electroporation was dependent on temperature when adding drugs 5 minutes before electroporation. Here treatment effect was significantly greater at 21 **°**C than at 17 **°**C (p<0.05).

With calcium electroporation and calcium-bleomycin electroporation an optimum in treatment effect was found at both 27 °C and 21 °C indicating that the transition of the HT29 plasma membrane in the presence of calcium took place at a temperature between 21 °C and 27 °C.

## Supplementary 3

(Supplementary to Figure 4)

## a) HT29 dot plot and quadrant statistics

## b) MDA-MB231 dot plot and quadrant statistics

## c) HDF-n dot plot and quadrant statistics

**Figure S3. Representative dot plots from cytometric analysis of three cell lines***a) HT29, b) MDA-MB231, and c) HDF-n untreated cells stained with Annexin-V-FITC and PI. Dot plots show the gating of the events (all cells except debris are selected) and the division of the events in four quadrants with positive and negative staining for Annexin V and PI. Below the dot plots percentages of cells in each quadrant is displayed. Annexin V+/PI- (quadrant LR) cells were considered positive for exposure of PS in the outer leaflet of their cell membranes.*
